# PPAI: a web server for predicting protein-aptamer interactions

**DOI:** 10.1186/s12859-020-03574-7

**Published:** 2020-06-09

**Authors:** Jianwei Li, Xiaoyu Ma, Xichuan Li, Junhua Gu

**Affiliations:** 1 Institute of Computational Medicine, School of Artificial Intelligence, Hebei University of Technology, Tianjin, China; 2 Tianjin Key Laboratory of Bioelectromagnetic Technology and Intelligent Health, Hebei University of Technology, Tianjin, China; 3 Tianjin Key Laboratory of Animal and Plant Resistance, College of Life Sciences, Tianjin Normal University, Tianjin, China

**Keywords:** Aptamer, Protein-aptamer interaction, Sequence features, Adaboost, Random forest

## Abstract

**Background:**

The interactions between proteins and aptamers are prevalent in organisms and play an important role in various life activities. Thanks to the rapid accumulation of protein-aptamer interaction data, it is necessary and feasible to construct an accurate and effective computational model to predict aptamers binding to certain interested proteins and protein-aptamer interactions, which is beneficial for understanding mechanisms of protein-aptamer interactions and improving aptamer-based therapies.

**Results:**

In this study, a novel web server named PPAI is developed to predict aptamers and protein-aptamer interactions with key sequence features of proteins/aptamers and a machine learning framework integrated adaboost and random forest. A new method for extracting several key sequence features of both proteins and aptamers is presented, where the features for proteins are extracted from amino acid composition, pseudo-amino acid composition, grouped amino acid composition, C/T/D composition and sequence-order-coupling number, while the features for aptamers are extracted from nucleotide composition, pseudo-nucleotide composition (PseKNC) and normalized Moreau-Broto autocorrelation coefficient. On the basis of these feature sets and balanced the samples with SMOTE algorithm, we validate the performance of PPAI by the independent test set. The results demonstrate that the Area Under Curve (AUC) is 0.907 for prediction of aptamer, while the AUC reaches 0.871 for prediction of protein-aptamer interactions.

**Conclusion:**

These results indicate that PPAI can query aptamers and proteins, predict aptamers and predict protein-aptamer interactions in batch mode precisely and efficiently, which would be a novel bioinformatics tool for the research of protein-aptamer interactions. PPAI web-server is freely available at http://39.96.85.9/PPAI.

## Background

Nucleic acid aptamers against proteins have attracted tremendous attention since they were discovered, the interactions between proteins and aptamers are one of hotspots of biochemistry, molecular biology, bioinformatics and biophysics [1]. Due to the high affinity and specificity of nucleic acid aptamers, protein-aptamer interactions have become more significant for targeted drug therapy of complex diseases and have a perform a variety of functions [2–5]. Aptamers are typically identified in vitro from random libraries of DNA or RNA molecules using an iterative process of Systematic Evolution of Ligands by Exponential Enrichment (SELEX) [6], which consists of several repeated rounds of binding, partition and amplification. The aptamers have the merits of easy synthesis and good stability, their specific bindings to proteins play an important role in various life activities of the organisms. Although the experimental aptamer screening technology has been further developed recently, it still has more disadvantages such as time-consuming, expensive and labor-intensive. For this purpose, effective computational methods for predicting aptamers and protein-aptamer interactions are urgent and necessary.

In recent several years, machine learning methods have been widely used in the prediction of protein-aptamer interactions, some computational models have been developed, for example, Li et al. [7] developed a random forest-based protein-aptamer interaction prediction model, Zhang et al. [8] presented a novel model based on the ensemble method in 2016. However, there are still certain limitations in above models. In the Li′s model, feature extraction of the sequences was relatively simple and it did not balance the training samples, which resulted in high prediction accuracy for large sample class and low accuracy for small sample class. The same datasets were adopted in Zhang’s model and it extracted different features based on the multiple feature extraction strategy and reconstructed training dataset. To reconstruct the training dataset, the positive and negative samples were split into three groups according to the ratio 1:1, each group was consisted of 580 positive samples and 580 negative samples. These three data sets were facilitated as training sets of three random forest models and the averaged results of the three random forest classifiers were accepted as the final prediction results. Zhang’s model also balanced the accuracy of large sample class (i.e. negative samples) and that of small sample class (i.e. positive samples), but the negative samples of each random forest classifier is less due to the split of training data, which led to a decrease of the overall accuracy.

In order to predict aptamers and protein-aptamer interactions more accurately, in response to the above problems, we improved the processing of building datasets and extracting more sequence features, integrated predictive capabilities of two machine learning methods, and developed a novel web server named PPAI. In our study, there was a prominent imbalance ratio between positive samples and negative samples which could lead to the inherent learning biases [9]. Therefore, the SMOTE [10] method was first to utilize to amplify small sample data for the unbalanced datasets, the balanced dataset could avoid biases in the machine learning. Moreover, PPAI also stores more known protein-aptamer interactions into its database for making user query at easy. In previous studies, more useful features based on structural and evolutionary information did not fully understand or used [7, 8]. Multiple useful features can preserve enough discriminative information for protein-aptamer interaction pairs [11], the combination of various features from different heterogeneous features is a good strategy for enhancing the performance and robustness of a predictor [12]. Based on the multiple feature extraction strategy, multiple key sequence features of proteins and aptamers were synthesized. After analyzing the unique secondary structure characteristics of the aptamers deeply, we screened negative samples and selected the optimal feature set for predicting aptamers. In general, an ensemble method that integrated diverse learning polices of multiple basic classifiers could outperform its component classifiers [13]. Therefore, an ensemble method combining the adaboost and random forest method was developed to predict protein-aptamer interactions in PPAI.

## Results

### The performance of protein-aptamer interaction prediction

**Fig. 1:**
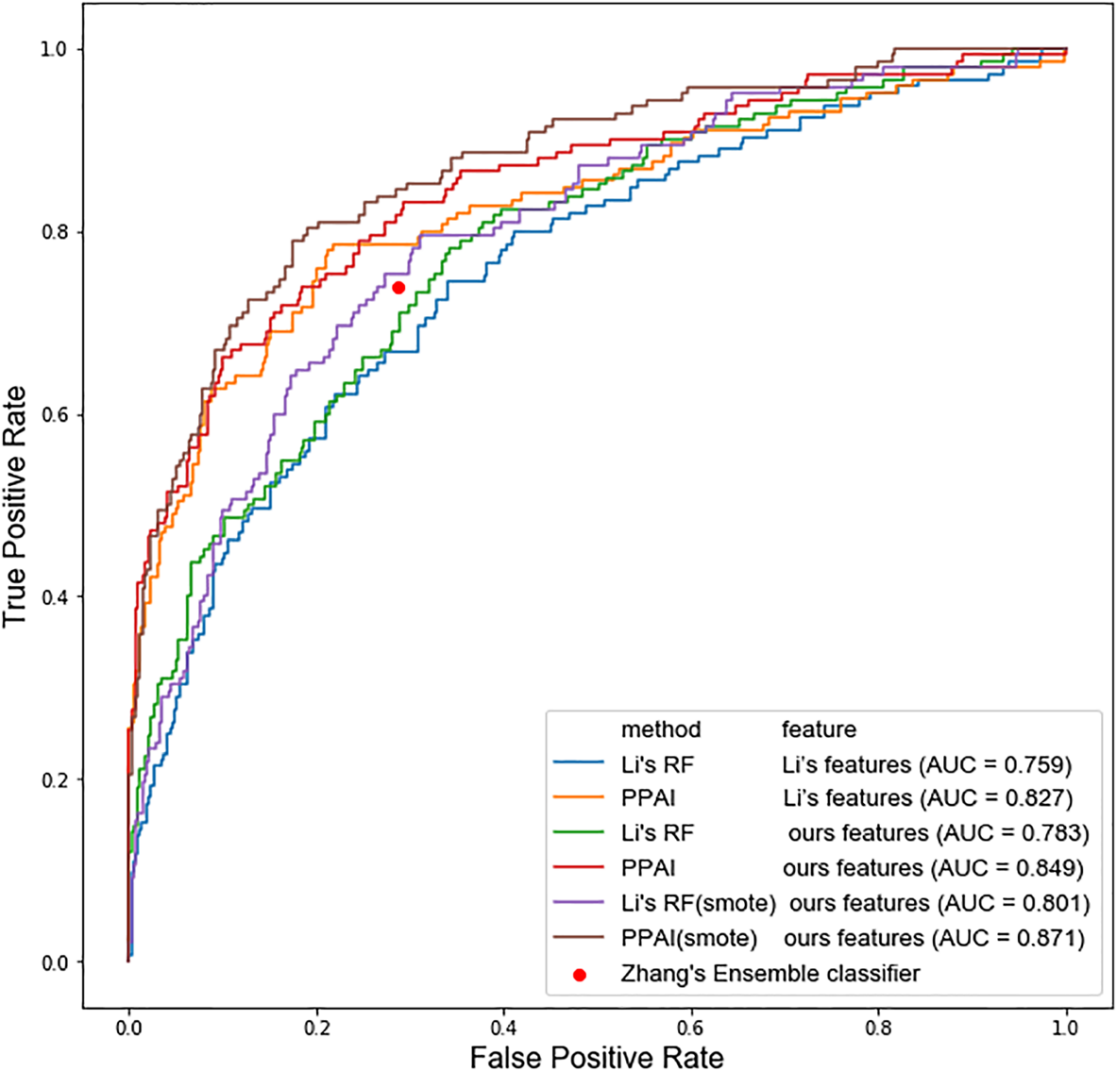
ROC curves illustrating the overall performance comparison results using different combination of machine classifier and features

Experiments were performed to show both the accuracy of our classifier and effectiveness of feature extraction in depth. The performance comparison based on the same dataset can reflect the performance of a predictor more reliably. To better evaluate performance of PPAI objectively, we compared PPAI with Li’s model on the same datasets, using various combination of features and machine learning classifier. The method and features of Li’s model given in the published article were utilized to repeat the experiment and reproduce the model. The Receiver-operating characteristics (ROC) curve was drawn through the reproduced model. The detailed prediction results are shown in Table 1 and Fig. 1. According to Table 1, PPAI has better predictive performance in terms of area under ROC curve (AUC) by comparing Li’s features versus ours features. After balancing training set with the SMOTE method, more balanced sensitivity and specificity were obtained. Moreover, better AUCs could be achieved after introducing SMOTE (Fig. 1). We have also adopted the statistical method to test whether such improvement of AUC should be significant by using the pROC package of R software. The result of statistical test has shown that while the improvement of Li’s method is not significant (DeLong’s test. *P* = 0.471), applying SMOTE indeed significantly enhance the prediction performance of our method (DeLong’s test, *P* = 0.0401). The ROC curve was drawn by plotting *Sn* versus *Sp* at different thresholds, it can more intuitively compare the performance of the above models [14]. Where both *F1* value and AUC (Area under ROC curve) are improved, the overall prediction performance is also improved. Furthermore, As the Zhang’s ensemble model has no tool or open source code, its threshold is fixed leading to a single point in Fig. 1 that corresponds to its performance in the ROC curve.

**Table 1:** Performance comparison using different combination of machine classifier and features

Method	*Sn*	*Sp*	*MCC*	*Acc*	*F1*	AUC
Li’RF (Li’s features)	0.483	0.871	0.372	0.774	0.517	0.759
PPAI (Li’s features)	0.572	0.924	0.538	0.836	0.636	0.827
Li’RF (ours features)	0.458	0.915	0.422	0.800	0.535	0.783
PPAI (ours features)	0.641	0.903	0.557	0.842	0.664	0.849
Li’RF (Smote) (ours features)	0.648	0.818	0.441	0.775	0.592	0.801
PPAI (Smote) (ours features)	0.796	0.810	0.555	0.806	0.675	0.871

Accuracy and AUC of our model reaches 0.806 and 0.871 respectively, both higher than two previous models. Moreover, our model obtains a more balanced performance with Sn (0.796) and Sp (0.810). To further explore the important features that contribute to the prediction performance, we also extracted the feature importance scores from the model. The top protein features and top nucleotide features are shown in Additional file 1: Supplementary Tables S1-S2, respectively. Notably, features from various encodings constitute the top feature set, emphasizing the importance of using multiple sequence feature encodings.

### The performance of aptamer prediction

**Fig. 2:**
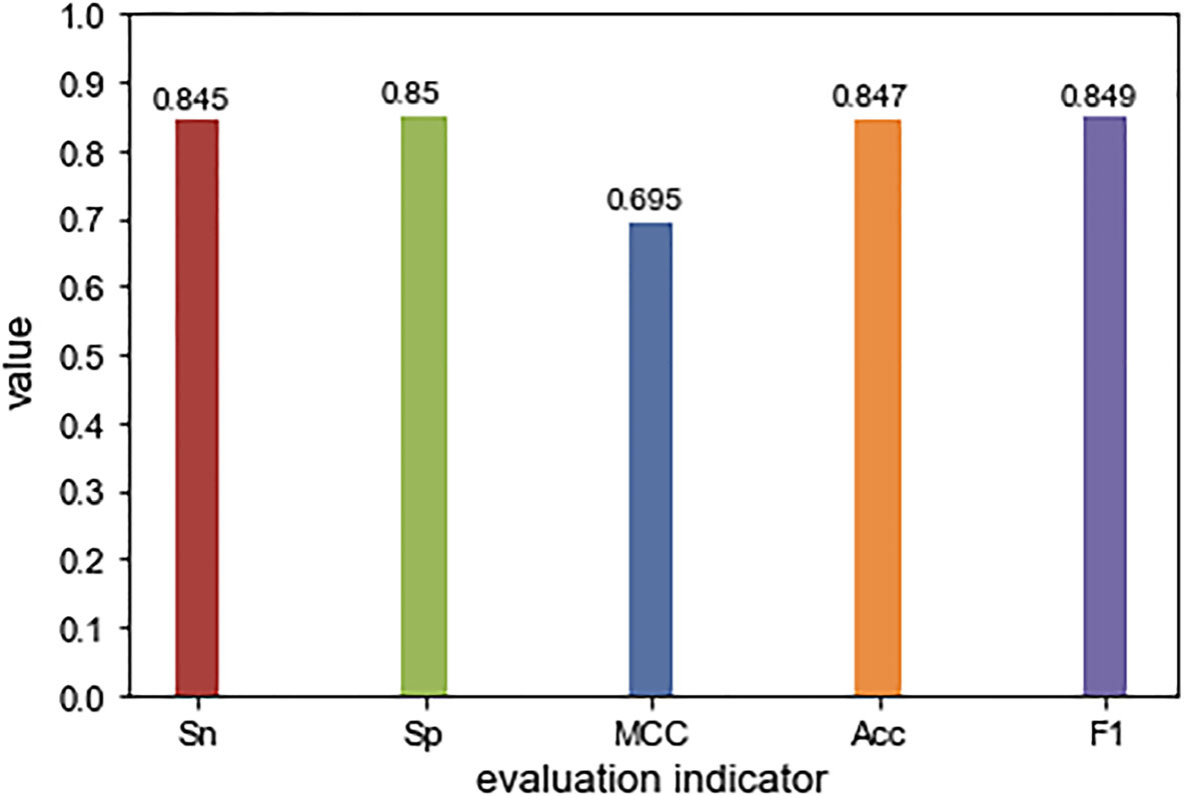
The performance of the aptamer prediction model

Besides predicting protein-aptamer interactions, PPAI offered function of predicting aptamers for nucleotide sequences that user input. In order to verify the performance of the aptamer prediction model, the experiment was conducted on an independent test set. To our best knowledge, we cannot find the similar method to be compared with it, so only the performance of PPAI was reported (see Fig. 2).

**Fig. 3:**
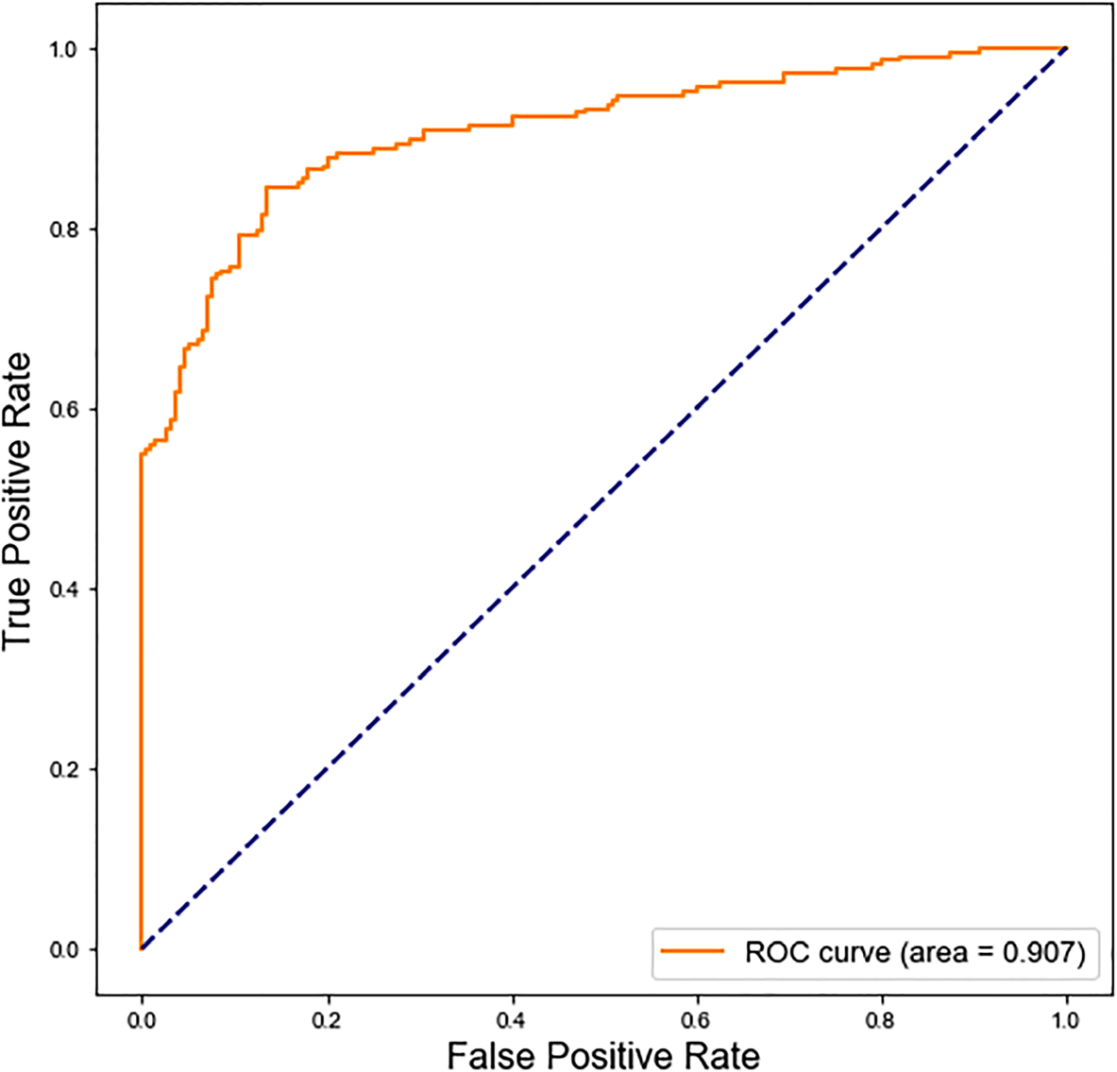
ROC curve of the model of aptamer prediction

As shown in Fig. 2, the accuracy on the independent test set reached 0.847, and the *F1* value reaches 0.849, which suggests the accuracy and practicability of the decision model constructed in this experiment. In order to more intuitively reflect the predictive performance of this model, ROC curve was drawn and the AUC was further calculated to evaluate the performance of aptamer prediction in the independent test (Fig. 3), where PPAI has achieved an AUC of 0.907.

### Overview of PPAI web server

In order to facilitate the community, we developed a web server named PPAI. Depends on user inputs, PPAI provides three major functions. First, if users input a name or a sequence of a protein/aptamer in the ‘Query’ page of PPAI, the query function is provided. After data collection, we all gathered 704 aptamers and 156 proteins data in PPAI for users to query (see Additional file 1: Supplementary Tables S3-S4). If users submit an aptamer name/sequence, the tabular result is firstly provided, which includes the number of protein-aptamer interactions and the associated proteins. For each associated protein in the tabular results, user can also click the interesting protein name to view its details from Uniprot database. User can also input a protein name/sequence, PPAI provides its detail information which involved gene, organism, recommended name, entry id of Uniprot, the number of aptamers and the corresponding aptamer names. For example, the user can enter the name or sequence of the aptamer ‘9,572,845-PKR protein-3′, and click the ‘submit’ button to get information about the aptamer. The result is shown in Additional file 2: Supplementary Figure S1. The user can further click on the name of the protein of interest in the ‘Protein’ column of the result table to get information about that protein. Second, for aptamer prediction, users can choose ‘Predict Aptamer’ page which supports prediction of aptamers from a sequence list of DNA/RNA. The sequences of DNA and RNA should be submitted separately because the threshold values for DNA and RNA feature calculation are different. For example, if the user submits nucleic acid sequences in FASTA format (here we use the sequences of several known aptamer and non-aptamers as the examples), PPAI will give a prediction result for each sequence. It should be noted that the submitted file name should not contain special characters such as “()”, “.” and so on, so as not to cause the program to encounter the file error. The prediction result contains three columns, which are the sequence name, the prediction result (‘yes’ represents that it is an aptamer, and ‘no’ represents that it is not an aptamer), and prediction score (the probability that the sequence is an aptamer). The example result is shown in Additional file 2: Supplementary Figure S2. Third, if users have both interesting protein sets and aptamer sets, protein-aptamer interaction prediction is enabled in the ‘Predict Pairs’ page. Users should submit the aptamer file and protein file separately, and PPAI will give a prediction for each possible interaction pairs between each aptamer and each protein. Here we used the aptamers 17,030,508-Bovinefactor-IX-1 and 9,452,437-oligoadenylatesynthetase-4, together with 9 proteins as the example input. The result is shown in Additional file 2: Supplementary Figure S3. Each prediction result includes aptamer name, protein name, prediction result and predicted score. It should also be noted that the predictions of DNA-protein pairs and RNA-protein pairs should be done separately, because the physicochemical properties of DNA and RNA are not the same during feature extraction. Furthermore, the submitted file name should not contain special characters such as “()”, “.” and so on, so as not to cause the program to encounter the file error. One key point for the successful determination of protein-aptamer pair was the selecting of an appropriate threshold. In the simulation experiment with 3000 random sample pairs, we found 0.44 is an optimum threshold to predict the true protein-aptamer pairs, users can also reset the threshold according to their own needs. In general, higher threshold will increase specificity but will also miss more true positives.

PPAI used MySQL database to store the datasets, and its interface was implemented by HTML and CSS. EasyUI framework was adopted to enhance the page load and response faster. The asynchronous submission and partial refresh mode of PPAI were realized with Jquery+AJAX. The scripting language was C#, both extraction of sequence feature and calculation of predicted score were performed by Python.

## Discussion

Through the comparison experiments of different machine learning algorithms and features, the effectiveness of the algorithm and feature space mentioned in this paper is proved. The model constructed in this study for protein-aptamer interaction prediction has higher accuracy and more balanced sensitivity and specificity compared with two previous models, suggesting that PPAI has a fairly good prediction performance in predicting protein-aptamer interactions. The ROC curves of each model in Fig. 1 more intuitively reflect that the prediction performance of the PPAI model is superior to other models. Besides, above results effectively demonstrated its potential ability of predicting aptamers, it was beneficial for understanding the functions of aptamers and improving aptamer-based therapies. In addition, based on the current situation of lack of tools for protein-aptamer prediction, a user-friendly PPAI system was developed that provides query functions, protein-aptamer interaction prediction functions, and aptamer judgment functions.

## Methods

### Datasets

In line with previous studies of predicting protein-aptamer interactions, we also downloaded the datasets constructed by Li [7] which adopted the data from Aptamer Base database [15] (see Additional file 1: Supplementary Table S5). It is the largest data set currently available, and it was adopted by most existing methods. Aptamer Base was a collaborative database including protein-aptamer interactions, detailed experimental conditions and reference literatures. The dataset was divided into a training dataset and an independent testing dataset in advance. We first discarded problematic data whose sequence contained B, N, or a mixture of U and T. For easy to compare, the same datasets were adopted in our study, the training set was composed by 561 positive samples, 1682 negative samples, and the test set contained 143 positive samples and 421 negative samples. The positive samples are the protein-aptamer pairs with interaction, and the negative samples are the protein-aptamer pairs without interaction. There was an extremely imbalance between the number of positive samples and the number of negative samples, which would cause biases in the machine learning [16]. Therefore, the SMOTE algorithm [10, 17] was employed to balance the samples in our study. In SMOTE algorithm, the oversampling of the small sample was not done by simply copying the known samples, but by synthesizing new samples according to the feature space which could solve the overfitting problem resulting by simple copy effectively. In order to ensure the validity of the prediction, the SMOTE method was only utilized to balance the training set, and the independent test set was solely consisted of real samples. After amplifying the small class samples, the training set for predicting the protein-aptamer interactions included 1681 positive samples and 1682 negative samples. In addition, because our models are based on the sequence information of aptamers and proteins, if there is sequence redundancy in the data set, it may cause biases in prediction performance. To check this, we have also used CD-HIT to remove redundant sequences (50% identity threshold for proteins and 80% identity threshold for nucleotides) in the dataset and re-analyzed the performance. The results suggest that the prediction performance is acceptable either before or after removing sequence redundancy, while the better performance of our method can be still observed (Additional file 1: Supplementary Table S6).

**Fig. 4:**
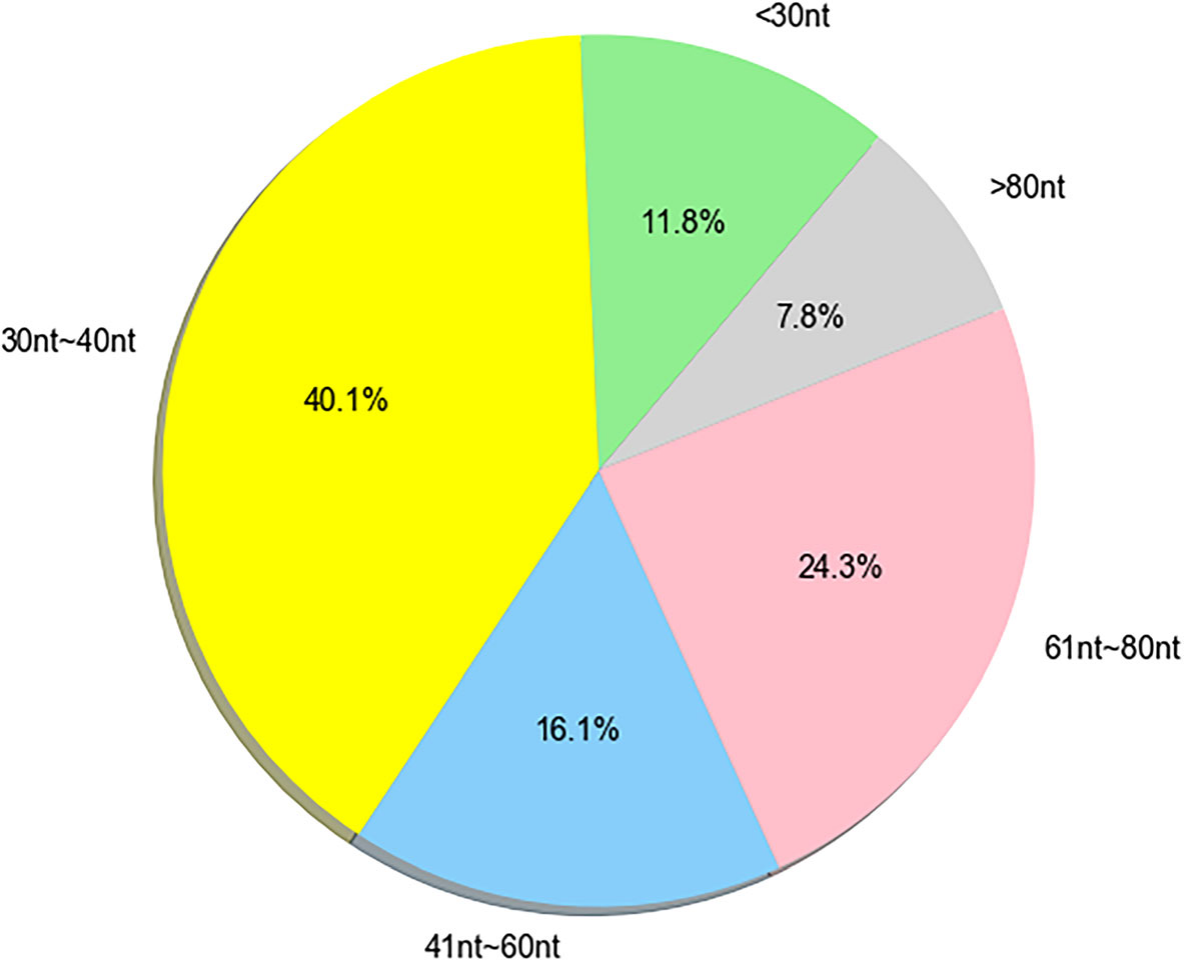
Length distribution of positive sample aptamer sequences

As for the prediction of aptamers, 704 positive samples and 700 negative samples (350 DNAs and 350 RNAs) were chosen as training dataset and independent testing dataset for predicting the aptamers (Additional file 1: Supplementary Table S7). The positive samples refer to known aptamers, and the negative samples refer to randomly generated nucleotide sequences that show highly distinct secondary structure characteristics compared with known aptamers. The most important difference between aptamers and common RNAs/DNAs was that aptamers were easily folded into a pseudoknot, and the stem-ring structures were mostly convex rings and circle rings. Aptamers often had a large contact area to specifically bind to the target molecule with high specificity [18]. In our study, the RNAfold [19] was utilized to predict secondary structure with randomly generated sequences, those sequences that did not conform to the secondary structure pattern of the aptamers were assigned as the negative samples. Because the accuracy of RNAfold’s prediction of secondary structure is about 70% ~ 80%, there might be false negative samples. In order to reduce false negatives as much as possible, the negative samples were screened based on both the minimum free energy and the secondary structure, which could effectively reduce the occurrence of false negative samples. The aptamer has a more stable structure, and its minimum free energy is smaller. The characteristics of the secondary structure of aptamers were fully analyzed, and negative samples were selected from various aspects such as stem-loop structure and number of unpaired bases. These distinctions of secondary structures were obvious and not likely to be confused between aptamers and non-aptamers. The distribution of the lengths of aptamers (DNA or RNA) was shown in Fig. 4, about 80% of the sequence lengths were between 30 nt and 80 nt, the most common aptamer lengths are 40 nt, 30 nt, 50 nt and 80 nt. Based on the above, the lengths of the aptamers in the generating 700 negative samples were according with the length assignments of the aptamers in the 704 positive samples.

### Feature extraction

Converting an input sample sequence into a set of numerical features is a crucial problem in designing a predictor. Previous studies [7, 8] had shown that pseudo-amino acids and pseudo-nucleotides were effective features for predicting protein-aptamer interaction pairs. The specific binding between aptamers and proteins is closely related to their respective physicochemical properties, which are crucial factors for their secondary structures [20]. The secondary structures of nucleic acid strands are the main and effective features for distinguishing the aptamers from the common nucleic acid strands. In general, a single feature extraction strategy can only represent partial samples’ characteristics, multiple feature extraction strategies can enhance the prediction accuracy. Based on above description, this study combined several key physicochemical features of proteins and aptamers in both the aptamer prediction and the protein-aptamer interaction prediction, and these features were calculated by the iFeature [21] package and the pseKNC [22] package, respectively.

Based on the large numbers of experiments, the considered features of proteins in our study included amino acid composition [23], pseudo-amino acid composition [24], grouped amino acid composition [25], C/T/D composition [26] and sequence-order-coupling number. Amino acid composition means the frequency that is the number of times that each amino acid occurred in the sequences composed by 20 kinds of amino acids. The pseudo-amino acid composition is originally proposed by Chou to predict protein properties [24]. Pseudo amino acid composition has been proved to be an effective feature for many biological problems [7, 27, 28]. Twenty kinds of amino acids are divided into 5 groups in grouped amino acid composition according to their physicochemical properties such as hydrophobicity, charge and molecular size. Each group is defined as

1 $$ f(g)=\frac{N(g)}{N},g\in \left\{g1,g2,g3,g4,g5\right\} $$

where g1, g2, g3, g4, g5 represent aliphatic group, aromatic group, positive charge group, negative charged group and uncharged group and *N(g)* is the number of amino acid in each group.

C/T/D is a pattern of amino acid distributions of specific structural or physicochemical properties in a protein or peptide sequence. C/T/D composition means the ratio of amino acids of specific nature to the total number of amino acids. The physicochemical properties of seven amino acids were used in this study, which were hydrophobic, standardized van der Waals volume, polarity, polarizable, secondary structure, positive/negative charge and solubility. Each attribute is further divided into 3 groups according to its property. The calculation of the attributes is defined as

2 $$ f(r)=\frac{N(r)}{N},r\in \left\{r1,r2,r3\right\} $$

Where *r1, r2, r3* represent polar, neutral and hydrophobic and *N(r)* is the number of amino acid type r in the encoded sequence.

Sequence-order-coupling number is the distance between two amino acids calculated by the physicochemical distance matrix of amino acids. The distance matrix used the physicochemical matrix of Schneider-Wrede [29] and the chemical matrix of Grantham [30]. The calculation of each distance matrix is defined as

3 $$ {f}_d={\sum}_{i=1}^{N-d}{\left({d}_{i,i+d}\right)}^2,d=1,2,3\dots \lambda $$

where *d*_*i,i + d*_ is the distance between two amino acids in a given distance matrix, and *λ* (default is 30) is the maximum distance of the amino acids.

On the other side, the features of aptamers were extracted from nucleotide composition, pseudo-nucleotide composition (PseKNC) and normalized Moreau-Broto autocorrelation coefficient [31]. The nucleotide composition is the frequency at which each nucleotide (A, C, G, T/U) appears in the sequence. The pseudo-nucleotide composition is a feature proposed based on the pseudo-amino acid composition. The DNA/RNA sequence is converted into a set of discrete values. The calculating method of the pseudo-nucleotide is described in reference [32]. The normalized Moreau–Broto autocorrelation (NMBAC) was proposed by Feng et al. [31] to predict membrane protein types. We used NMBAC to extract features from 11 physical and chemical properties (shift, slide, rise, tilt, roll, twist, stacking- energy, twist, entropy, free energy, hydrophilicity) for protein-aptamer interaction prediction.

### PPAI model based on integrated framework of adaboost and random forest

A novel model for PPAI was developed to predict aptamers and protein-aptamer interactions with a machine learning framework integrated adaboost [33] and random forest [34]. Adaboost combines multiple weak classifiers into the final strong classifier. It would update the sample weights according to each training sample while training. For the misclassified samples, the weights of them are increased, the training set will be trained iteratively. The weight of each weak classifier will be calculated according to the error rate, the higher the error rate, the smaller the weight. Finally, all weak classifiers are weighted and summed to obtain final classification results. Normally, the classification result of each sample is often determined by a classifier with a larger weight. The final model can be calculated from *h*_*t*_ and *α*_*t*_ using follow formula:

4 $$ H(x)=\mathit{\operatorname{sign}}\left({\sum}_{t=1}^T{\alpha}_t{h}_t(x)\right) $$

Where *h*_*t*_ is the basic classifier and *α*_*t*_ is the weight of it. Furthermore, *α*_*t*_ is calculated by *ε*_*t*_ which is the deviation of *h*_*t*_:

5 $$ {\alpha}_t=\frac{1}{2}\ln \left(\frac{1-{\varepsilon}_t}{\varepsilon_t}\right) $$

The adaboost classifier can often omit some unnecessary training data features and focus on key features [35]. Besides that, the other advantage of adaboost method is that the feature selection process can be omitted. The default basic weak classifier of adaboost algorithm is decision tree which has the shortcomings of the low accuracy and classification efficiency for multi sequence features of proteins/aptamers. However, random forest is a strong classifier that integrates multiple decision trees. In order to improve the performance of predictive model, we adopted adaboost in combination with random forest. The random forest was modified as the basic classifier of adaboost. Furthermore, it was found through experiments that the prediction performance of the protein-aptamer interaction was not satisfactory when adopting the adaboost method alone, while the prediction performance was greatly improved by utilizing the combination of adaboost and random forest, and was better than the current methods (Additional file 1: Supplementary Table S8). Moreover, the prediction performance of different machine learning algorithms (Bayes, SVM, decision tree, random forest, PPAI) were also compared with the same set of features and the same datasets. The results show that the prediction method proposed in this study (adaboost combined with random forest) has the best prediction performance (see Additional file 1: Supplementary Table S9). Adaboost algorithm was implemented with Python’s sklearn package. After parameter optimization, in terms of the parameters of the random forest, the number of trees adopted the value of 10, and the parameter of ‘max_depth’ was set to 150. The adaboost method here mainly has three parameters, namely base_estimator, n_estimators and learning_rate (which determines the end condition of iteration). These three parameters are set to ‘random forest’, 300 and 0.75 respectively.

**Fig. 5:**
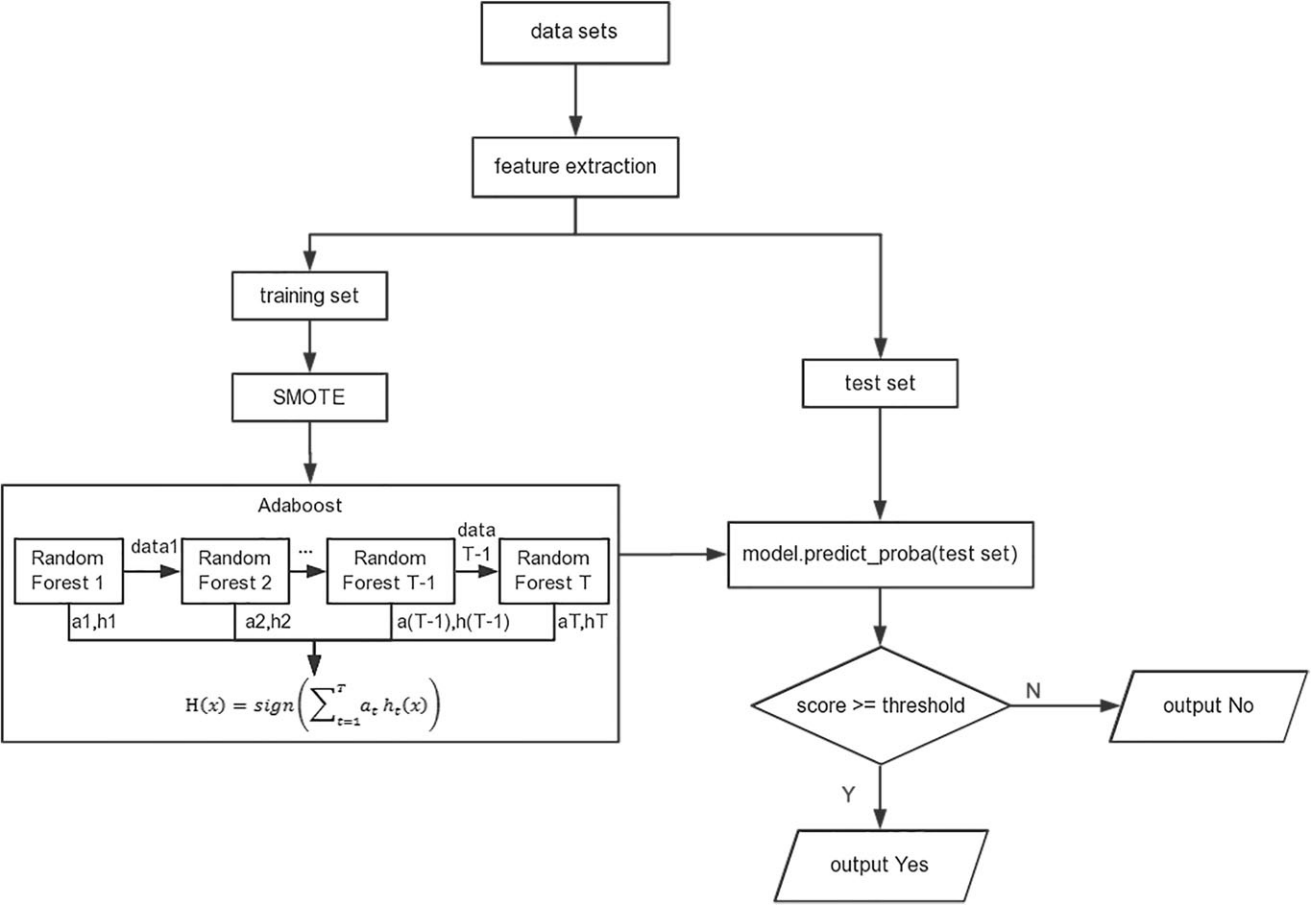
Flowchart of the prediction of protein-aptamer interactions in PPAI

The extracted feature vectors were used as input to train the model in PPAI, and the obtained model was tested with independent test dataset. The flowchart of protein-aptamer interaction prediction in PPAI is shown in Fig. 5. The predict_proba is as return value of the model, which is a real and presents the possibility of positive sample. Furthermore, we could adjust the threshold to optimize the prediction performance of the model. Setting threshold of predicting protein-aptamer interaction was to determine whether a protein and an aptamer interacted with each other. It was judged as ‘yes’ (interaction) when the score was greater than or equal to the threshold, and it was judged as ‘no’ (no interaction) while it was less than the threshold. The smaller the threshold, the higher the sensitivity (*Sn*) and the lower the specificity (*Sp*). When the threshold was 0.44, the *Sn* and *Sp* achieve the best balance, so 0.44 was set to the default threshold of predicting protein-aptamer interaction. In essence, aptamer prediction is a problem of binary classification like the prediction of protein-aptamer interactions. Therefore, the similar machine learning method was also adopted in the model. The threshold was set to determine whether the submitted sequence was an aptamer of one protein. By repetitious experiments, the best threshold was 0.48, and it was as the default threshold of predicting aptamer in PPAI.

### Performance evaluation criterion

The evaluation criteria of prediction performance adopted in this study were sensitivity (*Sn*), specificity (*Sp*), accuracy (*Acc*) and Matthews correlation coefficient (*M*CC). They are the most commonly utilized and basic evaluation index, which can show the prediction accuracy of positive and negative samples and the prediction accuracy of all samples, which can be defined as

6 $$ Sn=\frac{TP}{TP+ FN} $$

7 $$ Sp=\frac{TN}{TN+ FP} $$

8 $$ Acc=\frac{TP+ TN}{TP+ FP+ TN+ FN} $$

9 $$ MCC=\frac{TP\times TN- FP\times FN}{\sqrt{\left( TP+ FP\right)\left( TP+ FN\right)\left( TN+ FP\right)\left( TN+ FN\right)}} $$

where *TP, FP, TN* and *FN* represent true positive rate, false positive rate, true negative rate and false negative rate, respectively.

Moreover, because the testing dataset was unbalanced which would lead to a biased estimate of the accuracy, accuracy rate did not objectively evaluate the performance of the PPAI. Therefore, the weighted average of the precision rate and the recall rate (*F1*) is introduced as another criterion for performance evaluation, which is currently a widely used and effective evaluation standard for unbalanced data. It is defined as

10 $$ F1=\frac{2\times P\times R}{P+R} $$

where *P* and *R* are called Precision and Recall, respectively. While the *R* is equal to *Sn*, the Precision is defined as

11 $$ P=\frac{TP}{TP+ FP} $$

In the case of imbalanced data sets, the prediction accuracy is often biased toward the accuracy of the larger sample class (i.e. the negative samples for our cases), and cannot objectively reflect the prediction performance of the model. Therefore, ROC curve and AUC metric were introduced as the more appropriate evaluation criteria for such imbalanced dataset. The ROC curve can more intuitively compare the prediction performance of the models. The larger the area under the ROC curve (AUC), the better the prediction performance.

## Conclusions

It is important for biology research and drug design to accurately predict aptamers and protein-aptamer interactions by using various kinds of key sequence features of proteins and aptamers. In this paper, a novel ensemble method which is integrated with adaboost and random forest has been developed with a combination of various sequence features extracted from amino acid composition, pseudo-amino acid composition, grouped amino acid composition, C/T/D composition, sequence-order-coupling number, nucleotide composition, pseudo-nucleotide composition (PseKNC) and normalized Moreau-Broto autocorrelation coefficient to predict aptamers and protein-aptamer interactions. In order to solve the imbalance problem effectively, the SMOTE method was adopted to obtain balanced training datasets. To facilitate the community, a web server named PPAI was built with the abstracted sequence features and the machine learning framework mentioned above. PPAI has a user-friendly interface and step-by-step guide. The reliable performance of PPAI has been demonstrated in verification experiments with independent test datasets, we can draw a conclusion that PPAI is an efficient tool to predict protein-aptamer interactions which is better than the existing mainstream models. Comparing with other models, PPAI has two advantages: (1) More sequence features were introduced, which acquired more discriminative information for the predicitons; (2) The integration of adaboost and random forest, which results in a better performance. However, there exist some limitations, one major limitation is that the process of extracting sequence features is complex and time consuming, which is mainly caused by relative complex extracting algorithm. One solution is to improving things algorithmically and fixing inefficient code. Although limitations exist, we believe the PPAI provides aptamer researchers a valuable and efficient tool to predict protein-aptamer interactions.

## Supplementary information

**Additional file 1: Supplementary Table S1.** The feature importance scores of the top protein features of the model. **Supplementary Table S2.** The feature importance scores of the top aptamer features of the model. **Supplementary Table S3.** The information of aptamers. **Supplementary Table S4.** The information of proteins. **Supplementary Table S5.** The datasets of protein-aptamer interactions prediction. **Supplementary Table S6.** The performance comparison before and after removing redundancy of the dataset. **Supplementary Table S7.** The datasets for predicting aptamers. **Supplementary Table S8.** Performance comparison between using adaboost alone and using adaboost and random forest in combination. **Supplementary Table S9.** Comparison of prediction performance of different machine learning algorithms for predicting protein-aptamer interactions.

**Additional file 2: Supplementary Figure S1.** Example diagram of query module result of PPAI website. **Supplementary Figure S2.** Example diagram of predict aptamer module result of PPAI website. **Supplementary Figure S3.** Example diagram of predict protein-aptamer pairs module result of PPAI website.

## Data Availability

The datasets supporting the conclusions of this article are included with the article and its additional files.

## Abbreviations

PseKNC: Pseudo-nucleotide composition
AUC: Area Under Curve
SELEX: Systematic Evolution of Ligands by Exponential Enrichment
ROC: Receiver-operating characteristics
NMBAC: Normalized Moreau–Broto autocorrelation
SVM: Support Vector Machine
Sn: Sensitivity
Sp: Specificity
Acc: Accuracy
MCC: Matthews correlation coefficient

## Availability and requirements

Project name: PPAI

Project home page: http://39.96.85.9/PPAI (online webserver version)

Operating system(s): Platform independent

Programming language: Python, JavaScript, C#

Other requirements: Online webserver requires Chrome, Firefox or Microsoft Edge browser

License: FreeBSD.

Any restrictions to use by non-academics: None.
