# Supplementary material for: PPAI: a web server for predicting protein-aptamer interactions
Source: BMC Bioinformatics. 2020 Jun 9;21:236. doi: 10.1186/s12859-020-03574-7 (PMC7285591; doi:10.1186/s12859-020-03574-7)
Supplement: Supplementary file 2 — Additional file 2: Supplementary Figure S1. Example diagram of query module result of PPAI website. Supplementary Figure S2. Example diagram of predict aptamer module result of PPAI website. Supplementary Figure S3. Example diagram of predict protein-aptamer pairs module result of PPAI website. [file 12859_2020_3574_MOESM2_ESM.docx]

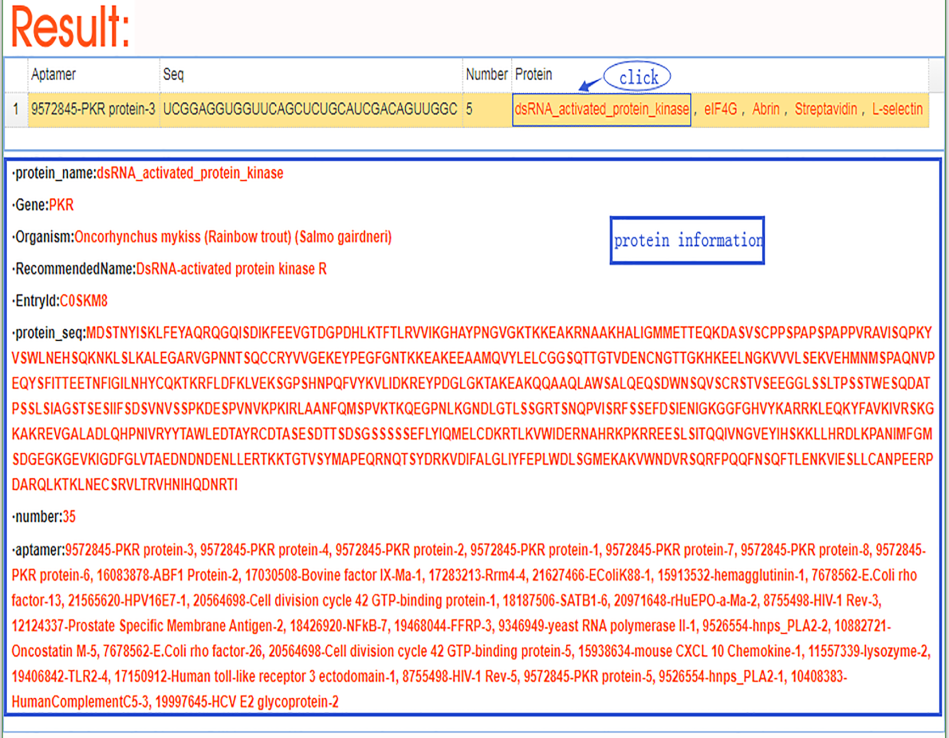


**Supplementary Figure S1.** Example diagram of query module result of PPAI website.


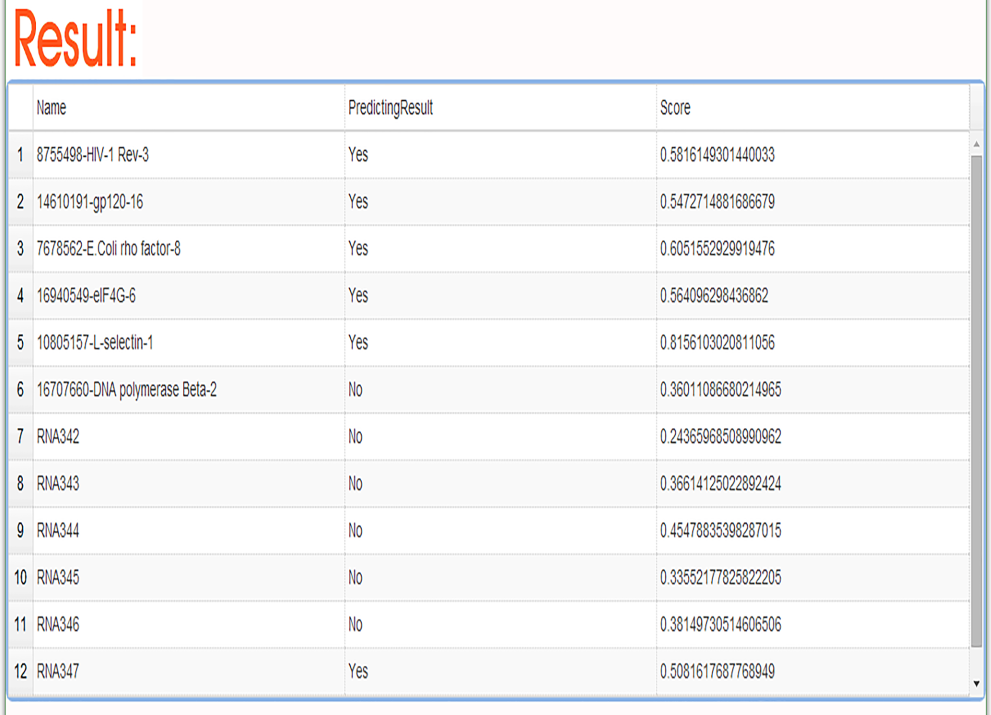


**Supplementary Figure S2.** Example diagram of predict aptamer module result of PPAI website.


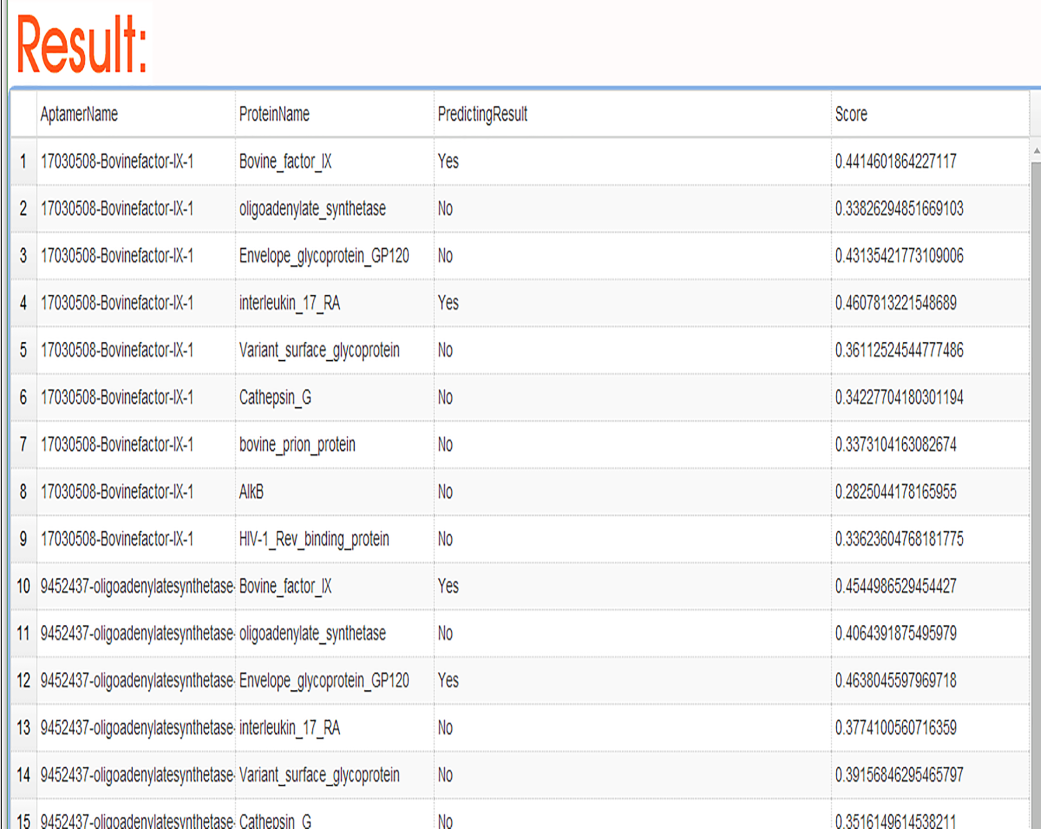


**Supplementary Figure S3.** Example diagram of predict protein-aptamer pairs module result of PPAI website.
